# *nm23-H1*基因转染前后人高转移大细胞肺癌细胞株的比较蛋白质组学研究

**DOI:** 10.3779/j.issn.1009-3419.2010.10.01

**Published:** 2010-10-20

**Authors:** 立伟 高, 文 朱, 潞 李, 梅 侯, 力 马, 颖 赵, 清华 周

**Affiliations:** 1 300052 天津，天津医科大学总医院，天津市肺癌研究所，天津市肺癌转移与微环境重点实验室 Tianjin Key Laboratory of Lung Cancer Metastasis and Tumor Microenvironment, Tianjin Lung Cancer Institute, Tianjin Medical University General Hospital, Tianjin 300052, China; 2 467000 平顶山，平煤集团总医院肿瘤内科 Department of Medical Oncology, Genernal Hospital, Affiliated to Pingmei Company, Pingdingshan 467000, China; 3 610041 成都，四川大学华西医院生物治疗国家重点实验室 Cancer center West China Hospital, Sichuan University, Chengdu 610041, China

**Keywords:** *nm23-H1*基因, 肺肿瘤, 双向凝胶电泳, 质谱, *nm23-H1* gene, Lung neoplasms, Two dimensional gel electrophoresis, Mass spectrometry

## Abstract

**背景与目的:**

肿瘤转移抑制基因*nm23-H1*基因调控肺癌细胞转移潜能的确切机理尚未明了，本研究旨在探讨人高转移大细胞肺癌细胞株（L9981）和转染*nm23-H1*基因的人高转移大细胞肺癌细胞株（L9981-nm23-H1）的差异表达蛋白，为阐明肺癌转移的分子机制、发现早期诊断肺癌转移的分子标志和新的治疗靶点提供实验依据。

**方法:**

应用固相pH梯度双向凝胶电泳分离L9981和L9981-nm23-H1细胞株的总蛋白，对25个差异明显的蛋白质点进行质谱鉴定和生物信息学分析。

**结果:**

研究观察到*nm23-H1*基因转染使人L9981细胞株蛋白质组的表达谱发生了明显变化：5个蛋白质表达缺失，9个新的蛋白表达，16个蛋白质表达下调，12个蛋白质表达上调。这些蛋白质主要涉及细胞骨架蛋白、信号转导蛋白、细胞代谢相关蛋白、发育增殖相关蛋白及肿瘤侵袭相关蛋白。

**结论:**

*nm23-H1*基因转染L9981后，蛋白质表达谱发生了显著的变化，这些差异蛋白质可能是逆转肺癌侵袭转移的生化基础，本研究结果可能为阐明肺癌转移的分子机制提供线索。

## Abstract (en)

**Background and objective:**

As a tumor metastasis suppressor gene, the functions of *nm23-H1* gene are still unclear. The aim of this study is to better understand the mechanism of lung cancer metastasis and to find new biomarkers for early diagnosis and new target for therapy by conducting comparative proteomics between the human high-metastatic large cell lung cancer cell lines (L9981) and L9981-nm23-H1 (constructed with transfecting *nm23-H1* gene into the L9981 cell line).

**Methods:**

The total proteins of L9981 and L9981-nm23-H1 were separated by immobilized pH gradient (IPG)-based 2-dimensional electrophoresis (2-DE); the significantly differently expressed proteins were examined by mass spectrometry and analyzed by bioinformatics.

**Results:**

It was observed that *nm23-H1* gene transfection caused remarkable changes of the proteome of L9981 compared with L9981-nm23-H1 cells: 5 proteins were deleted, 9 proteins appeared, 16 proteins downregulated, and 12 proteins up-regulated. These proteins are involved in cell framework, signal transduction, metabolism, proliferation and metastasis.

**Conclusion:**

After *nm23-H1* gene is transfected into L9981, proteome in L9981 is remarkably changed. These changes of the proteome could serve as a basis for reversing the invasive and metastatic phenotype in lung cancer and elucidating the machanisms of the metastasis of lung cancer.

肺癌侵袭转移是一个多步骤、多阶段、多因素和多基因控制的十分复杂的过程。阐明肺癌侵袭转移的分子机理、寻找和开发控制或逆转肺癌侵袭转移的新途径、新技术、新方法和新药物，是改善肺癌患者预后、提高生存质量和生活质量的最重要的措施。*nm23*基因是近年克隆并鉴定出的一种肿瘤转移抑制基因，该基因家族有8个成员，其中起主要作用的是*nm23-H1*基因^[1-7]^，本课题组从DNA、mRNA和蛋白水平就*nm23-H1*基因在肺癌中的表达水平变化、等位基因缺失与肺癌转移相关性等进行了一系列的研究，将这种多基因参与共同抑制肿瘤转移表型的作用称为“肺癌转移抑制级联”，提出*nm23-H1*基因可能是“肺癌转移抑制级联”中的一个关键基因^[1, 2]^。但是目前有关该基因调控肺癌细胞转移潜能的确切机理尚未明了，特别是其发挥作用的具体生化途径亦尚未明确，仍需更深入的研究。近年来蛋白质组技术由于高通量、高灵敏度的特点应用于肿瘤分子生物学研究。本研究采用比较蛋白质组技术，研究人高转移大细胞肺癌细胞株（L9981）与转染*nm23-H1*基因L9981细胞株（L9981- nm23-H1）间的差异表达蛋白，为阐明*nm23-H1*基因调控“肺癌转移抑制级联”的分子作用机制提供理论基础，为筛选逆转或控制肺癌侵袭转移的分子靶点及肺癌预后的分子诊断提供实验依据。

## 1 材料与方法

### 1.1 主要材料

#### 1.1.1 细胞株

L9981为四川大学华西医院四川省肺癌分子重点实验室应用单个细胞克隆化技术从人大细胞肺癌细胞株WCQH-9801中筛选建立的*nm23-H1*基因缺失的人高转移大细胞肺癌细胞株；L9981-nm23-H1为四川大学华西医院四川省肺癌分子重点实验室建立的转染野生型*nm23-H1*基因的L9981细胞株。

#### 1.1.2 主要试剂和仪器

丙烯酰胺、甲叉双丙烯酰胺、Tris-base、过硫酸铵、TEMED、二硫苏糖醇（dithiothreitol, DTT）、尿素、琼脂糖、溴酚蓝、CHAPS、SDS、低分子量标准蛋白以及24 cm IPG干胶条（pH3-10, 24 cm）均为瑞典Amersham Pharmacia公司产品；RNA酶（RNaseA）和碘乙酰胺为美国Sigma公司产品；甘氨酸及考马斯亮蓝为美国Amresco公司产品；所有溶液均用Milli-Q水配制。IPGphor水平电泳仪、垂直板电泳仪及图像分析软件ImageMaster^TM^ 2D Platinum Software（version 5.0）为瑞典Amersham Pharmacia公司产品；Daltonics AutoFlex TOFTOF LIFT Mass Spectrometer质谱仪购自德国Bruker公司。

### 1.2 方法

#### 1.2.1 细胞培养与细胞总蛋白制备

细胞传代培养于含10%小牛血清的RPMI-1640培养液中，生长至对数生长期时，胰酶消化，漂洗后将每2×10^6^个细胞重悬于100 μL预冷裂解缓冲液中，4 ℃静置30 min；冰浴下超声破碎细胞；4 ℃、20 000 rpm离心30 min，上清液即为所要的蛋白溶液；Bradford法^[8]^测定细胞总蛋白浓度，分装后-80 ℃保存备用。

#### 1.2.2 固相pH梯度双向凝胶电泳及凝胶图像分析

按照IPGphor^TM^等电聚焦系统操作指南，1 000 μg总蛋白与水化液混合均匀，总体积450 μL，用18 cm pH3-10的IPG胶条进行第一向水平等电聚焦和第二向垂直板SDS-PAGE电泳，凝胶经经典考染^[9]^置于扫描仪上扫描，得到数字化的凝胶图像文件。运用ImageMaster^TM^ 2D Platinum Software（version 5.0）图像分析软件进行分析，得到差异表达蛋白质点。实验重复3次。

#### 1.2.3 质谱鉴定及数据库查询

从凝胶上切取差异表达蛋白质点，脱色，胶内酶切，采用基质辅助激光解析电离飞行时间质谱（MALDI-TOF/TOF-MS）的方法，获得肽质量指纹谱，通过Mascot软件查询NCBInr数据库，肽片断序列匹配分数高于35分认为有统计学差异（*P* < 0.05）^[10, 11]^。

## 2 结果

对L9981与L9981-nm23-H1双向凝胶电泳图谱的蛋白点进行匹配、比较后发现：有5个蛋白点仅在L9981中出现，有9个蛋白点仅在L9981-nm23-H1中出现；在L9981与L9981-nm23-H1中均存在、但在L9981中的表达量显著高于L9981-nm23-H1的蛋白点有16个，在L9981-nm23-H1的表达量显著高于L9981的蛋白点有12个。L9981与L9981- nm23-H1间差异表达的蛋白点在凝胶上的分布见图 1、图 2。仅存在于L9981或L9981-nm23-H1中的蛋白点的放大图见图 3。

**1:**
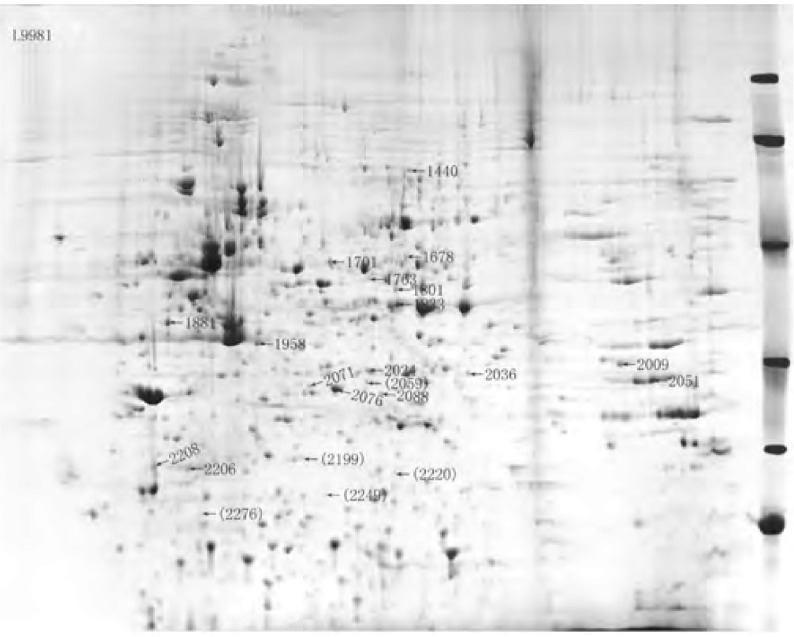
L9981与L9981-nm23-H1间差异表达的蛋白质点 Protein spots differentially expressed in L9981 compared with L9981-nm23-H1. Spot ID in bracket indicates the protein that was only detected in L9981; Spot ID no in bracket indicates the protein whose %vol in L9981 was more than two times higher than that in L9981- nm23-H1.

**2:**
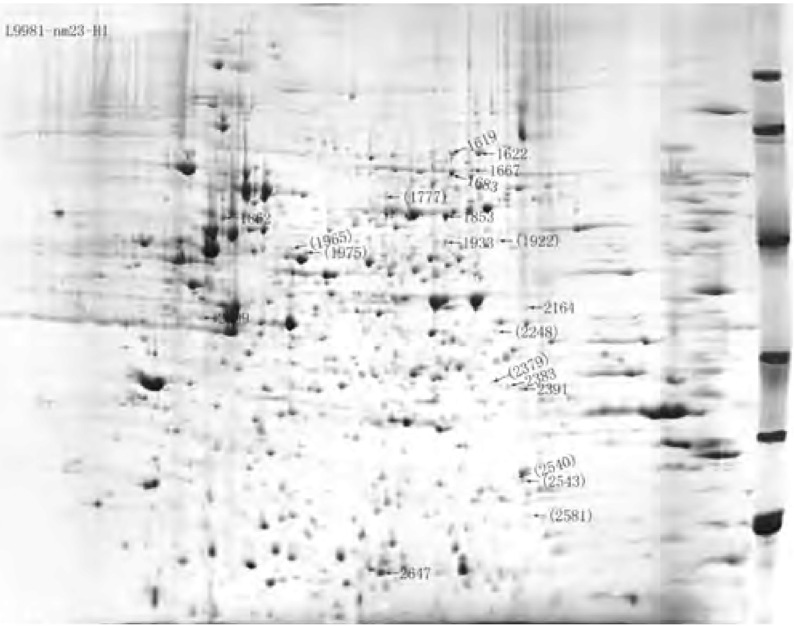
L9981与L9981-nm23-H1间差异表达的蛋白质点 Protein spots differentially expressed in L9981-nm23-H1 compared with L9981. Spot ID in bracket indicates the protein that was only detected in L9981-nm23-H1; Spot ID no in bracket indicates the protein whose %vol in L9981-nm23-H1 was more than two times higher than that in L9981.

**3:**
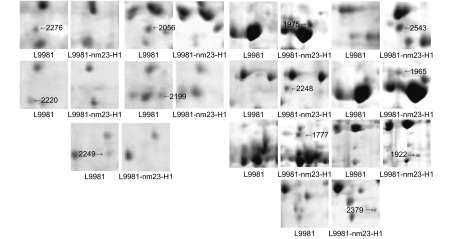
L9981与L9981-nm23-H1间部分差异表达的蛋白质点 Zoomed-in images of some differentially-expressed protein spots in L9981 and L9981-nm23-H1

从L9981与L9981-nm23-H1间差异表达蛋白点中选取25个差异明显的蛋白质点切割下来，胶内酶解，进行质谱鉴定，查询蛋白质数据库，获得17个L9981和L9981-nm23-H1间差异表达蛋白质的明确信息：*nm23-H1*基因转染使人高转移大细胞肺癌细胞株L9981原有蛋白-卤酸脱卤酶样水解酶包含域2（haloacid dehalogenase-like hydrolase domain containing 2）表达缺失；出现了三磷酸腺苷酶（ATPase）的表达；2-磷酸烯醇丙酮酸水合酶-α-烯醇酶（2-phosphopyruvate-hydratase alpha-enolase）、碳酸脱氢酶（carbonate dehydratase）、波形蛋白（vimentin）、甘氨酰tRNA合成酶（glycyl tRNA synthetase, GARS）、β-actin突变体、肽基脯氨酸异构酶D（peptidylprolyl isomerase D, PPIase D）、v-crk肉瘤病毒*CT10*癌基因类似同族体（v-crk sarcoma virus *CT10* oncogene homolog-like）、尿苷二磷酸葡萄糖脱氢酶（UDP-glucose dehydrogenase）、溶菌酶（lysosomal）、26S蛋白酶体调节链4表达下调，核纤层蛋白A/C转录异体1（lamin A/C transcript variant 1）、次黄嘌呤鸟嘌呤磷酸核糖基转移酶（HG phosphoribosyl transferase, HGPRT）、核纤层蛋白A、远端上游调控元件结合蛋白2、甲基丙烯酰-辅酶A-羧化酶2（methylcrotonoyl-coenzyme A carboxylase 2）表达上调。

## 3 讨论

迄今，有关*nm23-H1*基因转染前后人高转移大细胞肺癌细胞株L9981中蛋白质表达的变化，国内外未见报道。本研究将人高转移大细胞肺癌细胞株L9981和转染*nm23-H1*基因的人高转移大细胞肺癌细胞株L9981- nm23-H1三次实验的凝胶图像比较后发现，转染*nm23-H1*基因后使L9981蛋白质组的表达谱发生了明显变化：*nm23-H1*基因转染使人高转移大细胞肺癌细胞株L9981原有蛋白-卤酸脱卤酶样水解酶包含域2表达缺失；出现了三磷酸腺苷酶的表达；2-磷酸烯醇丙酮酸水合酶-α-烯醇酶、碳酸脱氢酶、波形蛋白、甘氨酰tRNA合成酶、β-actin突变体、肽基脯氨酸异构酶D、v-crk肉瘤病毒CT10癌基因类似同族体、26S蛋白酶体调节链4表达下调，核纤层蛋白A/C转录异体1、次黄嘌呤鸟嘌呤磷酸核糖基转移酶、核纤层蛋白A、远端上游调控元件结合蛋白2、甲基丙烯酰-辅酶A-羧化酶2表达上调。这些蛋白质大致可以归纳为5类：①细胞骨架相关蛋白：波形蛋白、核纤层蛋白A/C转录异体1；②信号传导蛋白：v-crk肉瘤病毒*CT10*癌基因类似同族体；③细胞代谢相关蛋白：卤酸脱卤酶样水解酶包含域2、2-磷酸烯醇丙酮酸水合酶-α-烯醇酶、碳酸脱氢酶、甘氨酰tRNA合成酶、肽基脯氨酸异构酶D、次黄嘌呤鸟嘌呤磷酸核糖基转移酶、甲基丙烯酰-辅酶A-羧化酶2、蛋氨酸腺苷转移酶Ⅱ，β-同工酶1属于蛋氨酸腺苷转移酶（methionine adenosyltransferase, MAT）家族；④发育、增殖相关蛋白：三磷酸腺苷酶、26S蛋白酶体（proteasome）调整链4、远端上游调控元件结合蛋白2（FUSE binding protein 2, FUSE-BP 2）；⑤肿瘤生长侵袭相关蛋白：尿苷二磷酸葡萄糖脱氢酶、溶菌酶。

ATP和ADO可作为P受体的激动剂刺激多种细胞的生长。Batra等^[12]^曾报道，低浓度ATP刺激SKOV-3肿瘤细胞增殖，高浓度ATP（100 μmol/L-1 mmol/L）则明显抑制SKOV-3的增殖。相反，Tey等^[13]^却发现，低浓度（10 μmol/L）ADO具有明显抑制A431肿瘤细胞增殖的作用，而高浓度的ADO（100 μmol/L）可促进其增殖。在本实验中我们观察到L9981-nm23-H1中出现了ATPase的表达，ATPase的主要功能为水解ATP，使L9981-nm23-H1肺癌细胞株内ATP浓度降低，我们的研究结果与Tey等的研究结果比较一致。推测低浓度ATP可能与抑制肿瘤的侵袭转移，ATPase可能是参与“肺癌转移抑制级联”的一种蛋白。

肿瘤细胞代谢以高比率的需氧糖酵解为其特征。这种高代谢需要相关的糖酵解酶的活性及其同工酶类型的相应改变以适应肿瘤的生物活性。催化糖原酵解途径中甘油分解的最后的酶—神经特异性烯醇化酶（neuronspecific enolase, NSE）研究较多，目前认为它不仅是所有类型神经细胞的标记物，而且是所有神经内分泌或神经旁细胞和各种各样的恶性瘤甚至非神经内分泌类型的标记物。有研究发现NSE在肾癌组织中的含量是正常肾皮质的34倍。80%的肾癌（renal cell carcinoma, RCC）患者NSE水平增高，且与肿瘤分期、分级呈正相关，与肿瘤的治疗随访、复发及转移有明显相关性^[14]^。本研究观察到转染*nm23-H1*基因后，人高转移大细胞肺癌细胞株L9981中2-磷酸烯醇丙酮酸水合酶-α-烯醇酶表达下调，提示2-磷酸烯醇丙酮酸水合酶-α-烯醇酶可能参与肺癌的侵袭转移过程。

波形蛋白（vimentin）曾作为间叶组织起源肿瘤的特异性标记。但近年来，文献^[15]^报道一些上皮性肿瘤也可以有波形蛋白表达。Catter等^[16]^报道肺鳞癌波形蛋白阳性率为30%，肺腺癌为23.5%。波形蛋白的阳性表达与肺癌患者的预后有明显联系。本研究观察到转染*nm23-H1*基因后，人高转移大细胞肺癌细胞株波形蛋白表达下调，提示该蛋白可能参与人大细胞肺癌的侵袭转移过程。

人酪氨酰-tRNA合成酶（tyrosyl tRNA synthetase, TyrRS）在血管发育的信号转导通路中起着非常重要的调节作用，可促进血管生成；而人色氨酰-tRNA合成酶的片段则具有抑制血管生长的功能。本研究观察到转染*nm23-H1*基因后，人高转移大细胞肺癌细胞株L9981甘氨酰tRNA合成酶（glycyl tRNA synthetase, GARS）表达下调，由此推断甘氨酰tRNA合成酶可能参与肺癌的侵袭转移过程。

核纤层蛋白A与C在细胞分化达到较高程度、显示一定表型的时候才出现。Broers等^[17]^对人肺癌细胞株进行研究，发现核纤层蛋白B在小细胞肺癌细胞株和非小细胞肺癌细胞株中均有表达；核纤层蛋白A和C在非小细胞株中表达，而在小细胞肺癌细胞株中表达极为低下或缺乏。较高水平的核纤层蛋白A和C反映了分化的、非增殖细胞的核稳定性。而低水平核纤层蛋白A和C是细胞核处于组织结构程度较低的细胞特征，表示细胞处于增殖周期过程。本研究观察到转染*nm23-H1*基因后，人高转移大细胞肺癌细胞株核纤层蛋白A/C（lamin A/C）转录异体1表达上调，提示该蛋白可能也是*nm23-H1*基因参与“肺癌转移抑制级联”的一种蛋白。

本研究结果为揭示*nm23-H1*基因肿瘤转移抑制的分子机制提供了理论基础和实验依据，可能有助于为逆转人大细胞肺癌细胞的侵袭转移提供新的治疗靶点。当然，由于双向凝胶电泳技术本身存在较多的局限性^[18, 19]^。本研究结果尚需进一步的深入研究，以证实这些差异表达蛋白在肺癌侵袭转移及“肺癌转移抑制级联”中的作用。
